# Radiomics Analysis of 3D Dose Distributions to Predict Toxicity of Radiotherapy for Cervical Cancer

**DOI:** 10.3390/jpm11050398

**Published:** 2021-05-11

**Authors:** François Lucia, Vincent Bourbonne, Dimitris Visvikis, Omar Miranda, Dorothy M. Gujral, Dominique Gouders, Gurvan Dissaux, Olivier Pradier, Florent Tixier, Vincent Jaouen, Julien Bert, Mathieu Hatt, Ulrike Schick

**Affiliations:** 1 Radiation Oncology Department, University Hospital, CHRU Morvan, 2 avenue Foch, CEDEX, 29609 Brest, France; vincent.bourbonne@chu-brest.fr (V.B.); omar.miranda@chu-brest.fr (O.M.); gurvan.dissaux@chu-brest.fr (G.D.); olivier.pradier@chu-brest.fr (O.P.); ulrike.schick@chu-brest.fr (U.S.); 2 LaTIM, INSERM, UMR 1101, Univ Brest, 29200 Brest, France; dimitris@univ-brest.fr (D.V.); florent.tixier@univ-brest.fr (F.T.); vincent.jaouen@univ-brest.fr (V.J.); julien.bert@univ-brest.fr (J.B.); hatt@univ-brest.fr (M.H.); 3 Radiation Oncology Department, Cornouaille Hospital, 29000 Quimper, France; dominique.gouders@ch-cornouaille.fr; 4 Clinical Oncology Department, Imperial College Healthcare NHS Trust, Charing Cross Hospital, Hammersmith, London W6 8RF, UK; d.gujral@nhs.net; 5 Department of Cancer and Surgery, Imperial College London, London SW6 4NE, UK

**Keywords:** radiomics, cervical cancer, toxicity, radiotherapy, NTCP

## Abstract

Standard treatment for locally advanced cervical cancer (LACC) is chemoradiotherapy followed by brachytherapy. Despite radiation therapy advances, the toxicity rate remains significant. In this study, we compared the prediction of toxicity events after radiotherapy for locally advanced cervical cancer (LACC), based on either dose-volume histogram (DVH) parameters or the use of a radiomics approach applied to dose maps at the voxel level. Toxicity scores using the Common Terminology Criteria for Adverse Events (CTCAE v4), spatial dose distributions, and usual clinical predictors for the toxicity of 102 patients treated with chemoradiotherapy followed by brachytherapy for LACC were used in this study. In addition to usual DVH parameters, 91 radiomic features were extracted from rectum, bladder and vaginal 3D dose distributions, after discretization into a fixed bin width of 1 Gy. They were evaluated for predictive modelling of rectal, genitourinary (GU) and vaginal toxicities (grade ≥ 2). Logistic Normal Tissue Complication Probability (NTCP) models were derived using clinical parameters only or combinations of clinical, DVH and radiomics. For rectal acute/late toxicities, the area under the curve (AUC) using clinical parameters was 0.53/0.65, which increased to 0.66/0.63, and 0.76/0.87, with the addition of DVH or radiomics parameters, respectively. For GU acute/late toxicities, the AUC increased from 0.55/0.56 (clinical only) to 0.84/0.90 (+DVH) and 0.83/0.96 (clinical + DVH + radiomics). For vaginal acute/late toxicities, the AUC increased from 0.51/0.57 (clinical only) to 0.58/0.72 (+DVH) and 0.82/0.89 (clinical + DVH + radiomics). The predictive performance of NTCP models based on radiomics features was higher than the commonly used clinical and DVH parameters. Dosimetric radiomics analysis is a promising tool for NTCP modelling in radiotherapy.

## 1. Introduction

Cervical cancer is the fourth most frequent cancer in women, with an estimated incidence of 70,000 new cases in 2018 [1]. Standard treatment for locally advanced cervical cancer (LACC) is chemoradiotherapy followed by brachytherapy. Compared with tri-dimensionnal external beam radiation therapy (EBRT), intensity-modulated RT (IMRT) can deliver an adequate dose to the target structures whilst limiting the dose delivered to pelvic and abdominal organs-at-risk (OARs), including bowel, rectum and bladder. This results in a lower incidence of early and late gastrointestinal and genitourinary (GU) toxicity [2,3].

Recently, 3D image-guided brachytherapy (IGBT) techniques have been implemented into clinical practice and allow for more favorable dosimetry and a reduced dose to nearby OARs, resulting in lower toxicity and improved local control [4,5]. Despite these advances, the toxicity rate remains significant [6] and these side effects can have an impact on long-term quality of life for these young patients [7,8].

Over the last decade, radiomics has emerged as a translational field of research, aiming at better characterizing medical images’ content via high-throughput extraction of engineered features [9,10,11,12,13]. Radiomics analysis has been applied to different imaging modalities, such as magnetic resonance imaging (MRI), positron emission tomography (PET), computerized tomography (CT) or ultrasound [14,15] to improve diagnostic and prognostic multiparametric models [14,16,17]. In radiotherapy (RT) applications specifically, radiomics has mainly been considered within the context of building models for the prediction of response to treatment and disease-free survival [13,18], whereas the prediction of toxicity endpoints has been determined by relying on, for instance, dose-volume histogram (DVH) metrics [19] and treatment-related clinical variables. Until now, the vast majority of studies have considered the application of the radiomics concept to the medical images, not dose distribution maps. Dose maps can also be considered as images with spatial and statistical distributions of dose levels and, therefore, can be analyzed through the radiomics approach with the aim of predicting toxicity endpoints [20].

The construction of models predictive of radiation-induced toxicity is of great interest to improve patients’ reported outcome quality of life and to facilitate possible radiotherapy dose escalation. In this study, we compared predictive modelling of toxicity events after RT for LACC, using the DVH parameters and radiomic features from dose maps.

## 2. Materials and Methods

### 2.1. Patients

Patients with histologically proven LACC stage IB1-IVA (FIGO 2009 definition) treated with definitive curative chemoradiotherapy (CRT) and subsequent brachytherapy (BT) between August 2010 and December 2016 at two French institutions (Brest and Quimper) were retrospectively included (Table S1, Supplementary Materials). Patients with stage IB1 and IIA1 were included if they had positive lymph nodes. All patients were required to have at least 3 years of follow-up. Exclusion criteria were history of previous chemotherapy or RT and/or metastatic disease.

Collected data included clinical characteristics, presence of positive lymph nodes on PET/CT, tumor size as measured on MRI, body mass index (BMI) before treatment, external beam radiotherapy (EBRT) and brachytherapy dose, presence of symptoms before radiotherapy (baseline), previous abdominal surgery, rectal comorbidities, date and status at last follow-up.

A total of 102 patients were included for retrospective analysis. The cohort was split into a training set (52 patients treated in Brest, 51%) and a testing set (50 patients treated in Quimper, 49%). All patients provided signed permission for the use of their clinical data for scientific purposes and informed consent for the anonymous publication of data. The Institutional Review Board approved this study (29BCR18.0015).

### 2.2. Treatment

Consortium guidelines were applied to contour the clinical target volume (CTV), the planning target volume (PTV) and organs at risk [21] (for bladder and rectum, we contoured the full volume). For brachytherapy, the vagina was retrospectively defined according to the method published by Susko et al. [22], as a 3-dimensional (3D) organ using a 0.2 cm fixed brush, expanded to include all vagina identifiable on MRI or CT images. The 0.2 cm brush was chosen because it was the smallest available from the treatment planning system.

All patients received pelvic EBRT or extended field RT to the para-aortic area using high-energy photons (18 MV), at a dose of 45–50.4 Gy with standard fractionation depending on their work-up. In patients with positive pelvic or para-aortic lymph nodes, an image-guided targeted boost was delivered to a dose of 50.4–54 Gy to the involved nodes (7 and 6 patients in training and testing sets, respectively). RT consisted of three-dimensional conformal radiotherapy (3D-CRT) (n = 33 and n = 32 in the training and testing sets, respectively) or IMRT (n = 19 and n = 18 in the training and testing sets, respectively) delivered using a linear accelerator.

Patients received 3–4 fractions of MRI-guided high-dose-rate (HDR) intracavitary brachytherapy every 4 days one week after EBRT completion. The prescribed dose was 7 Gy to the high-risk CTV. None of the patients had interstitial brachytherapy. No patient experienced delays or breaks in RT due to acute toxicity (median RT duration, 49 days; range, 47–53 days). All patients received 4–6 cycles of concomitant chemotherapy with weekly cisplatin (40 mg/m^2^) or carboplatin (area under the curve (AUC) 2) for those with renal impairment.

### 2.3. Parameters Used for Normal Tissue Complication Probability (NTCP) Modeling

To explore the possible value of dosimetric radiomics in predictive modeling, we focused on the incidence of GU, rectal and vaginal toxicities, using the Common Terminology Criteria for Adverse Events (CTCAE) v4. In the training set, the cumulative rates of acute (within 3 months of completing treatment) and late (later than 3 months after treatment) grade ≥2 GU, rectal and vaginal toxicities were 39%, 14%, 26%, 10%, 24%, and 24%, respectively. In the testing set, they were 42%, 16%, 28%, 14%, 24% and 24%, respectively.

- *Clinical Parameters*

Clinical parameters included in the analysis were the following: presence of symptoms before radiotherapy (baseline), age, FIGO stage, TNM staging, previous abdominal surgery, rectal comorbidities.

- *DVH Parameters*

Percentage and absolute volumes of rectum, bladder and vaginal receiving x Gy or higher (Vx), with x ranging from 5 to 55 Gy with EBRT and to 140 Gy with cumulate EBRT and BT doses, in 5 Gy steps, were included in the analysis. Dmean, Dmin, Dmax, D1cc, D2cc, D1% and D2% of rectum, bladder and vagina were also recorded for EBRT and BT doses. To account for the biological effects of different fractionation schemes, both the physical doses received with BT and EBRT were converted to EQD2 doses using a linear quadratic model [23] with an α/β ratio of 10 and 3 for dose summation for acute and late effects, respectively [24]. DVH from BT and EBRT were summed according to their relative volume and absolute dose, assuming that hotspots in the BT plan were located at the same location as hotspots in the EBRT plan. We recorded Vx_EBRT and Dx (minimum dose given to the hottest x% volume) EBRT for EBRT doses and Vx_EBRT + BT and Dx_EBRT + BT for cumulative EBRT and BT doses.

- *Radiomic Features*

For the purpose of this study, only the dosimetric map of EBRT was used. Doses were discretized according to the fixed bin width (FBW) method with a width of 1 Gy so that a minimum number of 32 bins would be obtained (max dose between 45 Gy and 55 Gy). For subsequent analyses, doses are denoted as “grey levels”.

In total, 91 image biomarker standardization initiative (IBSI)-compliant radiomic features (including first-order intensity metrics and higher order textural features) were extracted in 3D for each rectum, bladder and vagina dose distribution (i.e., volumes of interest corresponding to the manually delineated organs gross tumor volumes (GTV), reported on the corresponding dose maps). More information about feature implementation and benchmarking values can be found in the published literature [11,25,26]. All radiomics features are presented in Table S4 in the Supplementary Materials. Analyses were performed using in-house developed software (MIRAS v1.0, LaTIM, INSERM UMR 1101, Brest, France).

### 2.4. NTCP Modeling

For each toxicity endpoint, all generated NTCP models (including clinical features, DVH and radiomics alone or in association) were created with uni- and multivariate logistic regression. For each toxicity/parameter–set combination, the following workflow was performed to build the final multivariate logistic regression model: First, the features were selected by univariate analysis (*p* < 0.05) without correction for multiple testing in order to have at least a few parameters retained for building the models. The remaining significant features were further filtered based on Spearman’s rank test to exclude correlated parameters (>0.80), i.e., containing redundant information. If two or more features were correlated, the feature with the smaller *p*-value from the univariate analyses was retained. Multivariate logistic regression, with backward elimination, was then applied to determine the feature signature. All these steps were carried out using the training set only, for which the performance was evaluated by the AUC of the receiver operating characteristic (ROC) [27,28]. Based on the ROC obtained, the optimal cut-off values were selected in order to optimize accuracy (Youden Index).

The final signatures were evaluated on the testing set reporting balanced accuracy (BAcc) (calculated as the average of sensitivity and specificity) to identify patients with toxicity events. Balanced accuracy was used because of imbalance in the data [29].

All statistical analyses were performed using MedCalc Statistical Software version 15.8 (MedCalc Software bvba, Ostend, Belgium; https://www.medcalc.org; accessed on 10 December 2015). *p*-values below 0.05 were considered significant.

## 3. Results

For all toxicities, models tended to include more radiomic features (average of 1.33 ± 0.52 features) than clinical (average of 0.54 ± 0.29 feature) and DVH variables (average of 1 feature ± 0.55 features) (Table S2, Supplementary Materials).

The final models and the features included are reported in Table 1, Table 2, Table 3, Table 4, Table 5 and Table 6.

Model development in the training test: as presented in Table 1, Table 2, Table 3, Table 4, Table 5 and Table 6, the clinical features alone allowed AUCs_clinical_ of 0.53, 0.55, 0.51, 0.65, 0.56, and 0.57 for rectal, GU and vaginal acute and late toxicities, respectively. Considering only radiomic dosimetry features, the AUCs were higher for all toxicities compared to clinical parameters (AUC_RA_ of 0.72, 0.80, 0.81, 0.86, 0.79, 0.78 for the six endpoints, respectively), and always higher (except for GU late toxicities) than for DVH alone (AUC_DVH_ of 0.65, 0.78, 0.58, 0.57, 0.89, 0.72). For all toxicities (except GU acute and late toxicities), the predictive value of the models incorporating clinical and radiomic parameters was higher than that of the corresponding models based on clinical and DVH parameters. The differences in AUC were only statistically significant at the 0.05 level (*p* < 0.04) for acute and late vaginal toxicities.

The trend towards superiority of the models relying on radiomic dosimetry parameters was confirmed by the BAcc of the final models evaluated on the testing cohort, as reported in Table S3, Supplementary Materials. The addition of radiomic dose parameters to clinical + DVH models systematically improved their predictive value (i.e., BAcc_clinical_DVH_RA_ > BAcc_clinical_DVH_) except for GU acute toxicities. For late and acute vaginal toxicities, the difference was significant (*p* < 0.04). The best models combining all factors allowed us to reach a BAcc of 78%, 72%, 74%, 64%, 73%, and 78% for rectal, GU and vaginal acute and late toxicities, respectively.

## 4. Discussion

To the best of our knowledge, our work is one of the first studies investigating the use of radiomic features of dose distributions to predict toxicity endpoints in LACC. A previous study investigated rectal toxicities but did not investigate GU and vaginal toxicities [30].

Radiomics is the subject of an increasing number of medical studies [9,11,13,14,17,31]. In our study, we investigated a recently proposed use of radiomics in radiotherapy [20]. Indeed, for LACC specifically, we compared the predictive performance of NTCP models based on clinical parameters to commonly used DVH parameters of EBRT and brachytherapy, such as Vx and Dmean, and to radiomics features derived from 3D dose distributions, alone or in combination. Adding DVH parameters to clinical parameters systematically improved prediction performance. Furthermore, adding radiomic dose features resulted in incremental improvements for all tests (Table 1, Table 2, Table 3, Table 4, Table 5 and Table 6).

Our results in terms of DVH parameters are in accordance with previous reports, showing the V70% as predictive for late vaginal toxicity [32], and doses correlated to Dmax (D1cc, D2cc) as predictive for late rectal and GU toxicities [33,34].

DVH parameters commonly used in NTCPs, such as the OAR volume receiving more than a certain dose (V_D_) or Dmean, do not take into account information about the spatial relationship between voxels. Thus, the same V_D_ value can correspond to voxels distributed in the OAR or to connected voxels. But the biological impact of the same V_D_ may be different. In contrast, radiomic features explicitly take into account spatial relationships in the dose maps and are therefore expected to provide higher differentiating power.

To obtain more reliable and convincing results, we decided to evaluate the value of radiomic dose features in a framework of training and testing in different sets from two centers. Reproducibility is a recognized issue in the field of radiomics, especially with multicenter data [35]. For all data, we used the same dose calculation algorithm, implemented in 3D Slicer [36]. The objective of this study was to evaluate the added value of radiomic dose features in the building of NTCP models. We have detailed our methodological choices to achieve consistency in our approach. However, the robustness of the selected radiomic dose features with respect to variations in the dose calculation algorithm, grid size, and other factors was not evaluated in the present study and should be the subject of future research. Patient-specific variations between the expected dose and the administered dose could have a greater impact than the variations in calculated doses across dose calculation algorithms, the size of the grid applied, etc. To overcome the limitations of NTCP models based on DVH parameters, other dosimetric analyses were also published, such as the pixel-to-pixel comparison of the dose-to-surface map [37].

Two-dimensional dose surface analyses can also be performed with radiomics. Indeed, the frequency histograms of grey levels used in radiomics contain essentially the same information as dose-volume histograms. In this study, dosimetric parameters frequently used in NTCP models, such as Dmean and Vx, were included as DVH parameters, while Variance, Skewness and Kurtosis were considered as radiomic features.

We could have considered parameters such as Dmean and Vx to belong to the set of radiomic features. This approach could improve the predictive performance of NTCP models based on DVH parameters by including radiomics features.

Another method using radiomics to improve NTCP is based on characterizing healthy tissues from CT images through radiomic features which are then correlated with radiation-induced toxicity [38]. The majority of studies published so far focused on head and neck and lung cancers to predict the risk of xerostomia and radiation-induced pneumonitis [39,40,41,42,43]. The best models from these studies were based on the variation in radiomic parameters between pre- and post-treatment CTs. Thus, these models only allow one to predict toxicity at the end of treatment, whereas those derived from dose maps would allow for toxicity prediction at the time of treatment planning, enabling planning adjustments to decrease side effects.

We have followed a previously described methodology [17] consisting of the removal of features based on their correlation, in order to select a subset of radiomic features to be included in the statistical analyses. Models including more radiomics features could further improve the predictive performance of the models, which will require the use of more robust machine-learning techniques to select and combine parameters (e.g., embedded feature selection with Random Forest or neural networks). This will be the focus of future studies. Moreover, the relevance of the identified features will also be investigated for other tumor sites.

Our study has other limitations. First, the size of the two cohorts is small, but is explained by the low incidence of LACC in our country. The rate of chronic toxicity may also seem low, but is in accordance with what has been previously reported in the literature [44,45]. This resulted in limited statistical power that may contribute to explaining the lack of significance of some results, particularly with regards to chronic rectal toxicity. We did not apply correction for multiple testing when evaluating features in the training set, as none would have been significant if we did, and we had to reduce the number of variables by removing some of them based on their level of Spearman rank correlation. However, we mitigated the risk of false discovery by evaluating the trained models in a different, external cohort, which was not used in the training phase. The fact that the models performed well in the testing set increases the confidence in our findings and reduces the risk of overfitting when training them. These models, nevertheless, will require validation in a larger cohort.

Moreover, we only exploited the dosimetry map of external beam radiotherapy. The co-registration of external beam RT and brachytherapy treatment plans was difficult to achieve due to the presence of the brachytherapy device on the planning CT undertaken prior to brachytherapy [46]. In addition, patients treated with external-beam RT in Quimper underwent their brachytherapy treatment sessions in Brest, precluding the ability to carry out an external evaluation of the models. A potential solution could be to extract information from the brachytherapy dose map separately. However, at each brachytherapy session, a new dosimetry and a new dose map are performed, which poses the same problem of registration. Thus, although we did not perform radiomic analysis of the brachytherapy dose maps, the dose delivered by the brachytherapy was still taken into account by including the DVH parameters associated with the brachytherapy.

Organ motion can also impact the predictive value of radiomic parameters as well as DVH parameters. This is limited by the daily imaging control during treatment, allowing one to check the rectal repletion and the bladder filling.

Finally, it would be interesting to evaluate the combination of radiomic features extracted from images such as dosimetry CT with those derived from dose maps [38] in order to achieve the same endpoint prediction (both toxicity and tumor control or recurrence-free survival). However, this would entail handling an even larger set of features, which would probably require more complex modeling and larger patient cohorts. This will be the focus of future studies. Our models might achieve better performance by including the radiomic parameters extracted from the brachytherapy dose maps. However, they already perform better than the current models, and safe registration methods need to be validated before we can consider using the dose maps of the different brachytherapy sessions.

## 5. Conclusions

The 3D dose distributions provided by DVHs provide limited information, as the information about the spatial relationships between voxel doses is unavailable. The use of the radiomics approach applied to 3D dose distributions explicitly allows for taking this into account. This probably explains the higher predictive performance of NTCP models that include such radiomic dose features instead of common DVH parameters only. These findings highlight the need to go beyond EBRT planning-based DVH constraints, and dosimetric radiomics analysis could be a promising tool to personalize radiotherapy planning. However, the usefulness of the features described here will be investigated in prospective studies including a larger number of centers.

## Figures and Tables

**Table 1:** Results of the multivariate logistic analysis for acute rectal toxicity.

Rectal Acute					
Features	P	OR	10th–90th Perc. for OR	AUC	95% CI
Clinical • FIGO	0.66	1.14	0.32–5.72	0.53	0.39–0.67
DVH • V40%_EBRT	0.06	1.03	1.00–1.07	0.65	0.50–0.78
RA • Inv_diff_norm_cooc	0.030	9.61	3.59–133.99	0.72	0.58–0.83
Clinical + DVH • V40%_EBRT	0.04	1.04	1.00–1.08	0.66	0.51–0.79
Clinical + RA					
• Inv_diff_norm_cooc • FIGO	0.02 0.37	13.3 1.88	2.00–89.73 0.48–7.43	0.74	0.60–0.85
Clinical + DVH + RA					
• Inv_diff_norm_cooc • V45%_EBRT	0.04 0.30	63.51 1.01	5.91–104.96 0.99–1.04	0.76	0.61–0.86

OR = odds ratio, AUC = area under the curve, FIGO = International Federation of Gynecology and Obstetrics, V_x_ = percentage of rectum receiving x Gy or higher, DVH = dose-volume histogram, EBRT = external beam radiation therapy, RA = radiomic analysis.

**Table 2:** Results of the multivariate logistic analysis for acute genitourinary toxicity.

GU Acute					
Features	P	OR	10th–90th Perc. for OR	AUC	95% CI
Clinical • FIGO	0.55	1.42	0.39–6.31	0.55	0.40–0.69
DVH					
• V40%_EBRT • D1 Gy %_EBRT + BT	0.007 0.46	1.05 1.11	1.01–1.08 0.84–1.46	0.78	0.64–0.88
RA					
• Shade • ZSNU	0.036 0.29	1.00 0.99	1.00–1.01 0.99–1.01	0.80	0.66–0.89
Clinical + DVH					
• FIGO • V40%_EBRT • D1 Gy %_EBRT + BT	0.41 0.0041 0.40	2.04 1.07 1.15	0.38–11.03 1.02–1.12 0.84–1.58	0.84	0.71–0.93
Clinical + RA					
• ZSNU • FIGO	0.13 0.41	0.98 1.92	0.97–1.00 0.40–9.26	0.80	0.66–0.90
Clinical + DVH + RA					
• FIGO • V40%_EBRT • D1 Gy %_EBRT + BT • ZSNU	0.42 0.27 0.36 0.46	1.99 1.04 1.16 0.99	0.38–10.52 0.97–1.12 0.85–1.59 0.98–1.01	0.83	0.69–0.92

OR = odds ratio, AUC = area under the curve, FIGO = International Federation of Gynecology and Obstetrics, V_x_ = percentage of rectum receiving x Gy or higher, Dx minimum dose given to the hottest x% volume, DVH = dose-volume histogram, EBRT = external beam radiation therapy, BT = brachytherapy, RA = radiomic analysis.

**Table 3:** Results of the multivariate logistic analysis for acute vaginal toxicity.

Vaginal Acute					
Features	P	OR	10th–90th Perc. for OR	AUC	95% CI
Clinical • FIGO	0.51	1.35	0.41–6.14	0.51	0.37–0.66
DVH • D1 Gy %_ EBRT + BT	0.46	0.80	1.01–1.08	0.58	0.43–0.71
RA					
• HGLZE • LGSRE	0.048 0.057	1.00 1.34	1.00–1.01 0.58–1.85	0.81	0.68–0.91
Clinical + DVH • D1 Gy %_ EBRT + BT	0.44	0.79	0.44–1.43	0.58	0.43–0.71
Clinical + RA					
• HGLZE • LGSRE	0.048 0.053	1.00 1.34	1.00–1.01 0.58–1.85	0.82	0.69–0.91
Clinical + DVH + RA					
• HGLZE • LGSRE	0.048 0.053	1.00 1.34	1.00–1.01 0.58–1.85	0.82	0.69–0.91

OR = odds ratio, AUC = area under the curve, FIGO = International Federation of Gynecology and Obstetrics, V_x_ = percentage of rectum receiving x Gy or higher, Dx minimum dose given to the hottest x% volume, DVH = dose-volume histogram, EBRT = external beam radiation therapy, BT = brachytherapy, RA = radiomic analysis.

**Table 4:** Results of the multivariate logistic analysis for late rectal toxicity.

Rectal Late					
Features	P	OR	10th–90th Perc. for OR	AUC	95% CI
Clinical • FIGO	0.13	3.56	0.69–18.28	0.65	0.50–0.78
DVH • D1 Gy %_ EBRT + BT	0.34	1.19	0.84–1.68	0.57	0.43–0.71
RA					
• Coarseness vdif • AUC-hist	0.044 0.48	17.01 11.90	1.69–41.93 0.013–10.86	0.86	0.74–0.94
Clinical + DVH					
• FIGO • D1 Gy %_ EBRT + BT	0.14 0.39	3.41 1.17	0.67–18.21 0.82–1.68	0.63	0.48–0.76
Clinical + RA					
• FIGO • Coarseness vdif	0.069 0.020	5.99 11.71	0.87–41.13 1.54–89.35	0.87	0.74–0.95
Clinical + DVH + RA					
• FIGO • Coarseness vdif	0.069 0.020	5.99 11.71	0.87–41.13 1.54–89.35	0.87	0.74–0.95

OR = odds ratio, AUC = area under the curve, FIGO = International Federation of Gynecology and Obstetrics, V_x_ = percentage of rectum receiving x Gy or higher, Dx minimum dose given to the hottest x% volume, DVH = dose-volume histogram, EBRT = external beam radiation therapy, BT = brachytherapy, RA = radiomic analysis.

**Table 5:** Results of the multivariate logistic analysis for late genitourinary toxicity.

GU Late					
Features	P	OR	10th–90th Perc. for OR	AUC	95% CI
Clinical • FIGO	0.54	1.29	0.37–4.91	0.56	0.42–0.70
DVH • D1 Gy %_ EBRT + BT	0.0046	1.72	1.18–2.51	0.89	0.77–0.96
RA					
• Energy_hist • Inv_diff_norm_cooc	0.28 0.13	1.15 5.09	0.36–10.02 0.13–19.75	0.79	0.66–0.89
Clinical + DVH					
• FIGO • D1 Gy %_ EBRT + BT	0.38 0.0050	0.28 1.78	0.017–4.71 1.19–2.66	0.90	0.78–0.97
Clinical + RA					
• Energy_hist • Inv_diff_norm_cooc	0.28 0.13	1.15 5.09	0.36–10.02 0.13–19.75	0.79	0.66–0.89
Clinical + DVH + RA					
• D1 Gy %_EBRT + BT • Energy_hist • Inv_diff_norm_cooc	0.029 0.28 0.17	2.15 1.14 2.89	1.08–4.28 0.39–3.76 0.88–19.62	0.96	0.87–1.00

OR = odds ratio, AUC = area under the curve, FIGO = International Federation of Gynecology and Obstetrics, V_x_ = percentage of rectum receiving x Gy or higher, Dx minimum dose given to the hottest x% volume, DVH = dose-volume histogram, EBRT = external beam radiation therapy, BT = brachytherapy, RA = radiomic analysis.

**Table 6:** Results of the multivariate logistic analysis for late vaginal toxicity.

Vaginal Late					
Features	P	OR	10th–90th Perc. for OR	AUC	95% CI
Clinical • FIGO	0.38	1.82	0.47–6.96	0.57	0.42–0.71
DVH • V70 Gy%_EBRT + BT	0.015	1.30	1.05–1.60	0.72	0.58–0.84
RA • GLNU area	0.0097	0.93	0.87–0.98	0.78	0.64–0.88
Clinical + DVH • V70 Gy%_EBRT + BT	0.015	1.30	1.05–1.60	0.72	0.58–0.84
Clinical + RA					
• FIGO • GLNU area	0.39 0.01	1.94 0.92	0.42–8.89 0.87–0.98	0.79	0.65–0.89
Clinical + DVH + RA					
• V70 Gy%_EBRT + BT • GLNU area	0.013 0.0092	1.38 0.91	1.07–1.79 0.84–0.98	0.89	0.77–0.96

OR = odds ratio, AUC = area under the Curve, FIGO = International Federation of Gynecology and Obstetrics, V_x_ = percentage of rectum receiving x Gy or higher, DVH = dose-volume histogram, EBRT = External Beam Radiation Therapy, BT = Brachytherapy, RA = Radiomic Analysis.

## Data Availability

Not applicable.

## Supplementary Materials

The following are available online at https://www.mdpi.com/article/10.3390/jpm11050398/s1, Table S1: Patients’ characteristics, Table S2: Selection rate features, Table S3: Results in testing cohort, Table S4: List of radiomics features. For features detailed definitions and implementation, see Alex Zwanenburg, Martin Vallières, Steffen Löck: Image biomarker standardisation initiative—feature definitions. 2019. https://arxiv.org/abs/1612.07003; accessed on 26 March 2021.
