# Supplementary material for: Radiomics Analysis of 3D Dose Distributions to Predict Toxicity of Radiotherapy for Cervical Cancer
Source: J Pers Med. 2021 May 11;11(5):398. doi: 10.3390/jpm11050398 (PMC8151048; doi:10.3390/jpm11050398)
Supplement: Supplementary file 1 [file jpm-11-00398-s001.zip › jpm-1178919-supplementary.pdf]

Table 1: Patients' characteristics

	Brest		Quimper		Difference (p-value)
	n=52	%	n=50	%	
Age median (range)	57 (29-90)		58 (37-83)		0.84
FIGO stage					
IB1	1	2	2	4	0.52
IB2	4	8	7	14	
IIA	3	6	2	4	
IIB	30	60	21	42	
IIIA	1	2	0	0	
IIIB	7	13	7	14	
IVA	6	11	8	16	
Histology					
Squamous	40	77	40	80	0.90
Adenocarcinoma	9	17	6	12	
Adenosquamous carcinoma	3	6	3	6	
Clear cell carcinoma	0	0	1	2	
Lymph node involvment					
Uninvoled	26	50	25	50	0.84
Involved	26	50	25	50	
Pelvic	17	65	17	68	
pelvic and para-aortic	9	35	8	32	
CBC median (range)					
white blood cells	$6.5 \cdot 10^3$/mL (4.6-25.6)		$10.5 \cdot 10^3$/mL (4.9-22.7)		0.62
hemoglobin	128 g/dL (71-151)		123 g/dL (76-151)		0.61
platelets	$291.0 \cdot 10^3$/mL (171-819)		$266.0 \cdot 10^3$/mL (174-458)		0.48
body-mass index median (range)	21.5 (18-30)		25.5 (17-42)		0.23
Treatment					
3D-CRT	27	52	27	54	0.99
IMRT	25	48	23	46	
EBRT dose median (range)	45 (45-54)		45 (45-54)		1.00
BT dose median (range)	24 (21-28)		24 (21-28)		1.00
Overall treatment time (range)	49 (47-53)		49 (47-52)		0.

Abbreviations: FIGO= International Federation of Gynecology and Obstetrics, CBC= complete blood counts, 3D-CRT= three-dimensional conformal radiotherapy, IMRT= intensity-modulated photon radiotherapy, EBRT= external beam radiotherapy, BT=brachytherapy

Table 2: Selection rate features

Toxicity	Features	Selection rate
GI acute	Clinical	0.5
	DVH	1
	RA	1
GU acute	Clinical	0.75
	DVH	2
	RA	1
Vaginal acute	Clinical	0.25
	DVH	0.5
	RA	2
GI late	Clinical	1
	DVH	0.5
	RA	1
GU late	Clinical	0.25
	DVH	1
	RA	2
Vaginal acute	Clinical	0.5
	DVH	1
	RA	1

Table 3: Results in testing cohort

Toxicity	Features	BAcc	95% CI	AUC	95% CI
GI acute	Clinical	54.60	35.55-73.35	0.55	0.40-0.69
	DVH	55.59	38.80-69.25	0.56	0.41-0.70
	RA	78.41	57.3-92.15	0.78	0.65-0.89
	Clinical+DVH	52.14	36.75-65.25	0.52	0.38-0.67
	Clinical+RA	77.75	56.55-91.9	0.78	0.64-0.88
	Clinical+DVH+RA	78.41	57.3-92.15	0.78	0.65-0.89
GU acute	Clinical	55.36	35.3-76.4	0.55	0.41-0.69
	DVH	64.88	41.35-83.05	0.65	0.50-0.78
	RA	70.04	48.25-82.15	0.7	0.55-0.82
	Clinical+DVH	62.3	40.15-77.35	0.62	0.48-0.76
	Clinical+RA	55.56	40.00-63.05	0.56	0.41-0.70
	Clinical+DVH+RA	72.02	49.00-86.35	0.72	0.58-0.84
Vaginal acute	Clinical	53.51	33.40-75.85	0.54	0.39-0.68
	DVH	52.85	43.20-69.20	0.53	0.38-0.67
	RA	74.34	49.85-90.55	0.74	0.60-0.86
	Clinical+DVH	53.51	33.40-75.85	0.54	0.39-0.68
	Clinical+RA	74.34	49.85-90.55	0.74	0.60-0.86
	Clinical+DVH+RA	74.34	49.85-90.55	0.74	0.60-0.86
GI late	Clinical	55.66	33.25-80.8	0.56	0.41-0.70
	DVH	54.47	28.8-68.25	0.55	0.40-0.69
	RA	85.42	57.95-96.35	0.85	0.73-0.94
	Clinical+DVH	58.93	41.85-81.8	0.59	0.44-0.73
	Clinical+RA	65.18	44.5-87.0	0.65	0.50-0.78
	Clinical+DVH+RA	63.99	42.95-86.40	0.64	0.49-0.77
GU late	Clinical	66.94	39.90-89.15	0.67	0.52-0.80
	DVH	50.33	30.00-77.85	0.50	0.36-0.65
	TA	62.62	33.55-77.60	0.63	0.48-0.76
	Clinical+DVH	66.94	39.90-89.15	0.67	0.52-0.80
	Clinical+TA	69.60	39.90-84.20	0.70	0.55-0.82
	Clinical+DVH+TA	72.93	43.90-91.40	0.73	0.59-0.85
Vaginal late	Clinical	57.46	34.55-73.3	0.58	0.43-0.71
	DVH	67.55	43.1-86.30	0.68	0.53-0.80
	RA	76.10	52.45-87.9	0.76	0.62-0.87
	Clinical+DVH	63.82	39.30-81.75	0.64	0.49-0.77
	Clinical+RA	73.25	48.8-88.05	0.73	0.59-0.85
	Clinical+DVH+RA	78.29	54.25-93.40	0.78	0.64-0.89

Table 4: List of radiomics features. For features detailed definitions and implementation, see Alex Zwanenburg, Martin Vallières, Steffen Löck: Image biomarker standardisation initiative - feature definitions. 2019. https://arxiv.org/abs/1612.07003.

Class	Type	Method	Interpretation	main features
Shape	Geometric	3D descriptors	Geometric properties of the tumor volume and surface	Volume<br>Sphericity<br>Asphericity<br>Spherical disproportion<br>3D_surface<br>Ratio 3ds<br>Ratio 3d volume norm<br>Irregularity<br>Compactness 1<br>Compactness 2<br>Flatness<br>Elongation<br>Center of mass<br>Max 3D diameter<br>Least axis length<br>Major axis length<br>Minor axis length
Statistical	First-order	Histogram analysis	Global distribution of intensity values, in terms of spread, symmetry, flatness, uniformity and randomness.	Mean<br>Max<br>Min<br>P10<br>P90<br>Standard Deviation<br>Skewness<br>Kurtosis<br>Energy<br>Entropy<br>Variance
	Second-Order	Grey-level Co-occurrence Matrix (GLCM)	Spatial relationship between voxels in a specific direction, highlighting the properties of uniformity, homogeneity, randomness and linear dependency of the image.	Max<br>Entropy<br>Contrast<br>Dissimilarity<br>Variance<br>Average<br>Sum Average<br>Sum Variance<br>Sum Entropy<br>Difference average<br>Difference Variance<br>Difference Entropy<br>Angular Second Moment<br>Inverse Difference<br>Inverse Difference normalized<br>Inverse Difference moment<br>Inverse Difference moment

			normalized<br>Inverse variance<br>Correlation<br>Autocorrelation<br>Cluster tendency<br>Cluster Shade<br>Cluster prominence<br>Information correlation first<br>Information correlation second
Higher order	Neighborhood grey tone difference matrix (NGTDM)	Spatial relationship among three or more voxels, closely approaching the human perception of the image.	Complexity<br>Busyness<br>Contrast<br>Coarseness<br>Texture strength
	Grey-level Run-Length matrix (GLRLM)	Texture in a specific direction, where fine texture has more short runs whereas coarse texture presents more long runs with different intensity values.	Short-run emphasis (SRE)<br>Long-run emphasis (LRE)<br>Grey-level non-uniformity (GLNU)<br>Grey-level non-uniformity normalized<br>Run length non-uniformity (RLNU)<br>Run length non-uniformity normalized<br>Low Grey-Level Run Emphasis (LGRE)<br>High Grey-Level Run Emphasis (HGRE)<br>Short Run Low Grey-Level Emphasis (SRLGE)<br>Short Run High Grey-Level Emphasis (SRHGE)<br>Long Run Low Grey-Level Emphasis (LRLGE)<br>Long Run High Grey-Level Emphasis (LRHGE)<br>Grey-Level Variance (GLVAR)<br>Run-Length Variance (RLVAR)<br>Run percentage (RP)<br>Run Entropy
	Grey-level Size Zone Matrix (GLSZM)	Regional intensity variations of the distribution of homogeneous regions	Small Zone Emphasis (SZE)<br>Large Zone Emphasis (LZE)<br>Grey-Level Non-uniformity (GLNU)<br>Grey-level non-uniformity normalized<br>Zone-Size Non-uniformity (ZSNU)<br>Zone-Size Non-uniformity normalized<br>Zone Percentage (ZP)

		Low Grey-Level Zone Emphasis (LGZE)<br>High Grey-Level Zone Emphasis (HGZE)<br>Small Zone Low Grey-Level Emphasis (SZLGE)<br>Small Zone High Grey-Level Emphasis (SZHGE)<br>Large Zone Low Grey-Level Emphasis (LZLGE)<br>Large Zone High Grey-Level Emphasis (LZHGE)<br>Grey-Level Variance (GLVAR)<br>Zone-Size Variance (ZSVAR)<br>Zone size entropy
